# Ethics of participation and social inclusion of older persons in research: lessons learned from the COVID-19 pandemic in Singapore

**DOI:** 10.1186/s12961-022-00930-2

**Published:** 2022-11-29

**Authors:** Ad Maulod, Sasha Rouse, Atiqah Lee, Malcolm Ravindran, Hazirah Mohamad, Veronica Goh, Diyana Azman, Lian Leng Low, Rahul Malhotra, Angelique Chan

**Affiliations:** 1 Centre for Ageing Research and Education (CARE), Duke-NUS Medical School, Singapore, Singapore; 2 Department of Family Medicine and Continuing Care, Singapore General Hospital, Singapore, Singapore; 3 Independent Researcher, formerly affiliated to CARE, Singapore, Singapore

**Keywords:** Digital gap, Ageing, Vulnerability, Research methods, Ethics, Health services, Pandemic, Singapore

## Abstract

The COVID-19 pandemic brought about safe distancing measures that are unprecedented. This article details the impact of the pandemic across research studies involving older persons in Singapore, and assesses the different strategies used to adapt to their needs in the context of evolving public health measures. The pandemic exposed diverse experiences of vulnerability among older persons, pushing for critical reflections on ethics of participation and social inclusion as the new research normal. We emphasize the importance of practicing flexibility: consideration for differentiated approaches to recruitment and data collection that should be proactively embedded in research designs for older persons during the pandemic and beyond.

**Supplementary Information:**

The online version contains supplementary material available at 10.1186/s12961-022-00930-2.

On 7 February 2020, at the onset of the COVID-19 pandemic, Singapore raised its Disease Outbreak Response System Condition (DORSCON) alert level to Orange. The activation of DORSCON Orange stipulated the cessation of all hospital and community-based fieldwork requiring physical interactions until safety and control measures were put in place. On 7 April 2020, Singapore enforced a “Circuit Breaker” (CB)—a stay-home order and cordon sanitaire to control the outbreak. Since 19 June 2020, Singapore has begun easing its safety protocols in three transitional phases, each lasting for several months contingent upon the rate of disease transmission, leading to the eventual return to normalcy. As of February 2022, at the time this article was written, Singapore remains at DORSCON Orange, where limits in terms of social gatherings, group sizes and daily household visitors remain status quo. Eldercare centres and community social activities for older persons (aged 60 and above) were suspended in April 2020, but activities have gradually resumed since September 2020—albeit with restrictions. This resumption followed concerns about the adverse psychosocial impact suspending social activities had on older persons.

Adverse effects of the pandemic such as the higher risk of morbidity and mortality from COVID-19 infection on older persons have been widely established [1]. Another emerging and significant concern is the impact of physical distancing and isolation measures on older persons’ mental and physical health [2]. In Singapore, these measures resulted in the gradual physical and mental deconditioning of older persons [3]. Furthermore, as some services and social interactions shifted from the physical to the virtual to minimize physical contact, segments of older Singaporeans—particularly those with socioeconomic, health and/or digital constraints—struggled with the transition, laying bare the digital divide that exists even in a country that has been ranked one of the highest in the world for high-speed connectivity and internet affordability [4–6]. Despite numerous ongoing efforts by the Singapore government to improve digital uptake and literacy as well as providing social services that are within reach, the intensifying experiences of social isolation among older persons points to other important extenuating factors such as help-seeking behaviors, globalization, ageism and socioeconomic inequalities [7, 8]. Considering these factors, conducting research with certain segments of the older population became more challenging in the context of a pandemic because those individuals were difficult to approach physically and, in most cases, digitally.

Researchers have documented notable difficulties when conducting research face to face, resulting in many of such studies being prematurely discontinued [9]. Research methods that are adaptable, flexible and resilient have emerged as the new research normal [10]. As studies engage in alternative data collection methods to mitigate health risks and adhere to safe distancing restrictions, longstanding ethical and methodological concerns such as consent, rapport building, research inclusion and rigor remain in sharp focus. For example, Lobe et al. discussed the ethical issues surrounding obtaining virtual consent from individuals with limited digital competencies [11]. Additionally, poor Internet connectivity issues can hinder meaningful research participation or contribute to missing data [12], which compromises data robustness [13]. In longitudinal cohort studies that depend heavily on the quality of the relationships between researchers and respondents, preserving the cohort for future data rounds and taking steps to minimize respondent attrition and respondent fatigue becomes a surmountable challenge [14].

Studies conducted on alternative research methods during the pandemic have shown that despite the challenges, researchers have reconfigured research methods to ensure that research could continue, including among vulnerable populations such as older persons [11, 15]. This is an especially important consideration since older persons have lower participation rates in research studies in the first place [16]. However, there remains a significant literature gap in understanding the complexities of conducting research with older persons, with consideration given to the unique challenges posed during this pandemic.

In this article, we highlight seven studies involving older persons in Singapore, carried out by all coauthors at our centre (Centre for Ageing Research & Education, Duke-NUS Medical School Singapore) that were significantly impacted by safe distancing measures during the pandemic. We outline the challenges we encountered when research activities were first suspended and then subsequently resumed, and detail the strategies we undertook to mitigate risks of infection as well as project non-completion. We assess the feasibility of alternative modes of recruitment and data collection and explain why these may not work in the context of older persons in Singapore. We discuss how we adapted our research methods, particularly recruitment and data collection, to accommodate the needs of vulnerable older persons in Singapore—strategies that should continue to be implemented beyond the pandemic context. Our discussion outlines our critical reflections of conducting research with older persons during the pandemic, including what it means to design research that accommodates different types of vulnerabilities among older persons to ensure their voices are not excluded from research.

## Older persons in Singapore: understanding needs and research contexts

As of 2021, 16% of Singaporeans are aged 65 years or older, with recent estimates suggesting an increase of that proportion to 23% by 2030 [17, 18]. At the same time, Singaporeans are spending a greater proportion of their life span in poor health [17], with loneliness being associated with higher mortality risks [19]. Increased rates of chronic disease and disability among older persons have intensified eldercare demands in Singapore—where cultural discourses of filial piety places family caregivers as the major source of care [20]. The closure and restriction on eldercare services during the pandemic added a layer of complexity to the work of caregivers who were struggling to cope with existing stressors [21].

Despite the “inevitable” shift towards digitalization of healthcare catalysed by the pandemic [22], digital uptake among older Singaporeans remains a concern. Digital device utilization tends to decline dramatically with age. Yet this decline is even more pronounced for digital device utilization specific to health-related purposes [17]. Older persons who lack digital literacy or equipment may not be able to partake in social activities that have moved online in response to physical distancing and isolation measures during the COVID-19 pandemic, much less access digital healthcare services and systems. This could impact their health and psychosocial well-being.

Language also stood as a potential barrier for accessing healthcare and social participation, especially since English has been adopted as a medium of communication in these sectors. Only 35% of older persons in Singapore (above 65 years of age) are effectively bilingual (i.e. speaks both English and mother tongue). This limited access is more pronounced amongst older persons who have little or no formal education [17]. Stark differences in education levels are evident within different cohorts of older persons; 41% of those born between 1950 and 1959 did not complete high school education compared to only 8% for those born between 1970 and 1979 [23].

Poverty rates in Singapore increased by 43.5% from 2012 to 2015, with poverty levels in old age increasing by 74.3% in the same period [24]. Despite employment, poverty was observed to increase drastically for employed older persons [25], who are mostly concentrated in low-paying occupations [26, 27]. The lack of financial adequacy at retirement may result in downward class mobility, with some older persons having to sell their homes and take up rental housing to meet their basic needs [28]. With a significant proportion of older persons being diagnosed with two or more chronic illnesses [17], public assistance schemes are vital for those who are unable to work. Yet, these schemes operate via complex means-testing processes that exclude older persons who do not meet their strict criteria. The pandemic has caused disruptions resulting in sharp declines in income, rising unemployment rates and widening inequalities, bringing about catastrophic outcomes for older persons who were already in a precarious position [29].

While COVID-19 has impacted our research activities, it also further exacerbates the challenges faced by older persons in Singapore. Our experiences provide insights into the data collection strategies feasible during a pandemic, tailored to the appropriate contexts and needs of older persons in Singapore.

## COVID-19: impact on research activities in Singapore

Various pandemic-based nationwide restrictions that were implemented between February 2020 and March 2022 had significant impact on research activities at the centre, particularly participant recruitment, and data collection. In some months, our research had to be suspended until COVID-19 research safety protocols were put in place, and when they were resumed, in-person research activities had to adhere strictly to safety protocols that limited group sizes and number of household visitors (refer to Additional file 1: Table S1). As the measures regularly changed in response to the evolving pandemic situation, research activities had to be swiftly adjusted accordingly. Research teams had to reassess challenges and risks of disease transmission as they devised strategies to mitigate risks (see Table 1).

**Table 1:** Disease transmission risk and mitigation strategies for conducting interviews using different modes during the pandemic

Task	Challenges and risk of disease transmission	Strategies to mitigate risks
Scheduling interviews	Over the phone (low risk) – Researchers are informed when participants may have had a change in COVID-19 status, without physical contact In person (high risk) – Home visits required scheduling of interviews with referred participants who are not contactable – No way to determine whether participant is an active COVID-19 case prior to visit, or issued a health risk warning (HRW)	Over the phone – Call to schedule interview 7–12 days prior to intended interview date – Remind participants of scheduled interview 2–3 days prior and conduct COVID-19 safety checks – Call again on the day of interview and conduct COVID-19 safety checks – Reschedule interviews (14 days later) for those who are active COVID-19 cases, or a close contact In person – Mask-on all times, practice hand hygiene – Ring doorbell/knock on door and then stand 1 m away when waiting for a response – Conduct COVID-19 safety checks. If safe to enter, sanitize hands prior to entering household, offer participant a surgical mask and practice safe distancing
Conducting in-depth interviews remotely	Videoconferencing (low risk) – Challenge to obtain virtual/verbal consent due to diverse profiles of older persons – Access to internet connection that can support videoconferencing platforms	Videoconferencing – Digital consent forms available for tech-savvy participants via hyperlink – For participants who can do videoconferencing but do not know how to navigate digital consent (e.g. e-signatures), verbal consent must be sought prior to recording. Verbal consent will be audio-recorded – All recordings to be saved and uploaded to an encrypted cloud server and the local copy deleted
Conducting in-person data collection: in-depth interviews; photo elicitation interviews; participant observations; focus group discussions	In person (medium to high risk) – Participants may unknowingly be asymptomatic COVID-19 cases	In person – Mask-on always for both interviewer and participant, practice hand hygiene and safe distancing – Interview venue must be well ventilated – Ensure participant’s hands are sanitized before handling any items (e.g. documents, pen, tablet) – Sanitize all common touchpoints after each interview (e.g. pen, tablet) – For households, ensure participants will not expect more than the stipulated amount of unique household visitors after accounting for the interviewers

In addition, all interviewers must:

- Be fully vaccinated against COVID-19, and have received their booster shots;
- Take weekly antigen rapid tests (ART);
- Have an active contact-tracing application that is used to check in to places when necessary; and
- Ensure that those present for the interview are not under any COVID-19 restriction order or have had contact with COVID-19-positive cases within the past 10 days.

To continue conducting research in this pandemic, researchers must adapt to the constantly changing and unpredictable nature of these restrictions and build research resilience that can withstand resulting uncertainties.

## Unique challenges at different stages of fieldwork and with diverse older adult populations during the pandemic

Because of the diverse nature of our projects, recruitment and data collection challenges vary across projects. Table 2 outlines the different recruitment and data collection methods across seven projects that were ongoing at our centre during the pandemic.

I. Recruiting older persons in acute care settings

**Table 2:** Summary of research conducted during the pandemic

Project title	Profile of older adult participants	Recruitment methods prior to the pandemic	Recruitment methods during the pandemic	Data collection methods prior to the pandemic	Additional data collection methods during the pandemic	Changes to study timeline due to the pandemic
Study A* Mixed methods longitudinal evaluation on transitional care (*n* = 150)	– Frequently readmitted older persons (aged 50+) – Lower income bracket, living in public rental housing – No cognitive impairment; can answer independently	– Inpatient recruitment at the hospital wards; on-site recruitment	– No change	– Face-to-face interviews	– Online – Voice call – Videoconferencing	– 12-month hold in data collection – 12-month study extension granted
Study B* Longitudinal qualitative evaluation of an intergenerational befriending programme (*n* = 20)	– Older persons (aged 50+) who have been identified by community providers as socially isolated – No cognitive impairment; can answer independently	– Referrals from community providers – Door-to-door knocking	– Recruitment concluded prior to pandemic	– Face-to-face interviews	– Voice call – Videoconferencing	– 6-month hold in data collection – 6-month study extension granted
Study C Qualitative study on care integration for older persons (*n* = 95)	– Older persons (aged 50+) receiving two or more services from a community provider – Lower income bracket, living in public rental housing – No cognitive impairment; can answer independently	NA	– Referrals from community providers – Phone recruitment	NA	NA	NA
Study D Qualitative evaluation of a functional screening programme (*n* = 120)	– Older persons (aged 50+) who have undergone a nationwide screening programme – No cognitive impairment; can answer independently	NA	– Referrals from regional healthcare systems – Phone recruitment – On-site recruitment with strict COVID-19 safety protocols enforced	NA	NA	NA
Study E Qualitative study on social isolation on loneliness (*n* = 80)	– Older persons (aged 60+) – No cognitive impairment; can answer independently	NA	– Referrals from a previous national survey (participants who consented to being re-contacted for similar future studies) – Phone recruitment	– NA (study not started)	– Face-to-face interviews – Voice call – Videoconferencing	– 6-month extension to account for limited manpower due to COVID-19, and research limitations stemming from nationwide safe management measures
Study F* Longitudinal quantitative study on caregiving (*n* = 1085)	– 588 care recipients (aged 75+) who have received human assistance for their activities of daily living/instrumental activities of daily living (ADLs/IADLs) – May have cognitive impairment; may receive assistance to answer – 497 current or potential caregivers (aged 50+) – No cognitive impairment; can answer independently	– Referrals from two previous national surveys of older persons in Singapore (participants who consented to being re-contacted for future studies) – Letter of invitation sent to place of residence – Door-to-door knocking – Phone recruitment	– Phone recruitment	– Face-to-face interviews Offered on ad-hoc basis: – Online – Voice call	– Videoconferencing – Mailed-in survey Offered to all: – Online – Voice call	– 3-month hold in data collection – 18-month study extension granted
Study G Longitudinal qualitative study on caregiving (*n* = 60)	– Older persons (aged 50+) who are caregivers to other older persons (aged 50+) – No cognitive impairment; can answer independently	NA	– Referrals from Study F (participants who consented to being re-contacted for similar future studies) – Phone recruitment	NA	NA	NA

*Studies that were in progress when the pandemic began

NA: not applicable

Our mixed methods evaluation on transitional care (Study A) provides a good example to illustrate the complex interplay of factors affecting recruitment in acute care settings during the pandemic as recruitment was ongoing prior to the onset of the pandemic.

Prior to the pandemic, recruitment was done in person at the hospital wards of one public acute hospital in Singapore, with the research team having unrestricted access to the wards. Researchers could visit patients who were admitted and referred by hospital staff to take part in the research. During the pandemic, visiting protocols at the hospital were revised frequently to minimize community transmission of the virus and ensure patients’ safety (refer to Additional file 1: Table S2).

Uncertainties surrounding the duration of the pandemic resulted in unexpected recruitment difficulties for our research study. For example, when hospitals limited their healthcare services to minimize overcrowding and ensure that critical resources and manpower were dedicated to support ongoing pandemic-related operations [30], the programme we were evaluating was deprioritized, resulting in a decreased pool of eligible participants for Study A. Additionally, research teams, as nonessential staff, could not access hospital wards, while healthcare professionals were being redeployed to support pandemic operations and were not able to assist in recruitment. This adversely affected recruitment rates for Study A.

Even before the pandemic, language differences were already a major impediment to recruiting older Singaporeans for research [17, 31]. Having a team of recruiters with proficiency in different local languages contributed to recruiting a diverse sample pool. During the pandemic, however, we were limited to a single recruiter who was conversant in English and Mandarin, hence affecting the diversity of our sample pool.

II. Recruiting older persons in the community

In contrast to acute care settings, a different, less stringent set of measures apply in the community, making it easier to recruit from community partners without compromising nationwide COVID-19 safe management measures. Most of our studies (Studies A, B, C, E, F and G) rely on referrals from community partners, study sites or our research centre’s pre-existing database. Study D, which evaluates older persons’ participation in functional screening programmes, requires insights from participants about their experience with the programme. However, functional screening programmes were suspended since the “Circuit Breaker” in April 2020 and only resumed in early 2022. Study D was originally designed as a longitudinal study, collecting insights from participants recruited at baseline (first screening session) to completing intervention. However, due to COVID-19 disruptions, the entire study was reconfigured to a cross-sectional design. Recruitment is now primarily referral-based, corresponding to different intervention stages, with on-site recruitment employed as an alternative. To minimize physical contact, staff at the screening results station will introduce Study D briefly to the older persons, using a script we provided, after going through their screening results. Older persons who express interest will be ushered to a designated area for our research team to proceed with recruitment.

To minimize physical contact and the risk of infection transmission, phone recruitment became the default practice during the pandemic. Ethical consent requires referral parties (e.g. community providers, research teams, regional healthcare systems) to first obtain consent from the older person before transferring their contact details to our team.

However, phone recruitment was presented with a unique set of challenges as older persons in Singapore grew wary of accepting calls from unknown numbers in response to the emergence of widely reported fraud schemes that, exploiting heightened existential and financial anxieties, brought about significant financial loss [32]. Typically, our calls were ignored, adding to the existing fieldwork challenges brought about by the pandemic. To mitigate call rejections, extra steps were taken to prove our credentials and remove us from the possibility of identity fraud (e.g. providing detailed description about the study, university affiliations and intent to call). Despite having to furnish more details, we had to limit our phone calls to under 3 minutes to avoid using up prepaid phone credits for those with limited financial means.

Most participants recruited from acute care settings and the community preferred to have in-depth interviews (qualitative studies) conducted in person as they understood that the nature of the interview (e.g. duration, participatory component) would benefit from physical interactions. In fact, even with suggestions to conduct interviews/surveys remotely, most older persons (Studies A, B, C) who were approached declined participation because they did not have digital literacy or access. Older persons from these studies occupy the lowest percentile of household income in Singapore. They tend to be on welfare assistance and cannot afford mobile phones and data plans.

In addition, participants who were anxious about contracting COVID-19 would decline participation to avoid lengthy interactions despite our teams’ reassurances of adherence to safe management measures. For this reason, rejection rates during the pandemic were significantly higher in Studies A and B when compared to pre-pandemic.

III. Response from collaborators and funding agencies

Our funders and collaborators understood the challenges of doing research with older persons during the pandemic and acknowledged the importance of continued support for research activities without compromising the safety and well-being of research subjects and the research team. Most studies granted a no-cost extension of between 6 and 18 months, depending on the duration of fieldwork that was suspended or disrupted. Those who were not able to grant extensions provided our teams with additional support, such as ramping up participant referrals for recruitment, or allowing the research team to alter the research design (i.e. from a longitudinal study design to a cross-sectional study (Study D), reduce sample size or change study deliverables in the event of study non-completion.

IV. Feasibility of alternative modes of data collection

Participants across both the quantitative and qualitative studies expressed a strong preference for in-person interviews due to its convenience and more interactive nature. Participants declined alternative modes for conducting interviews (e.g. online, voice/video call, mailed-in survey) due to (a) health issues (e.g. digital amputation, sensory impairment, inability to look at a screen for prolonged periods), or (b) lack of digital literacy and/or smart devices or Internet access.

In our quantitative study (Study F), we received numerous incomplete submissions or “don’t know” responses when surveys were administered online or mail-in, mostly because skip-logic questions were too complicated to comprehend. The team had to contact participants to either verify their responses or complete the survey. Phone-in surveys were difficult to complete due to the lengthy duration and participants’ reluctance to answer calls from unknown numbers due to high incidence of phone scams (refer to Additional file 1: Table S3).

Conducting interviews remotely was less probable for our qualitative research, which typically focuses on poor older persons living in public rental flats who are in poor health (see Table 2; refer to Additional file 1: Table S4). We had difficulties re-contacting such participants for follow-up interviews (Study A)—they have either moved home or their phone lines were no longer active. We managed to get hold of only 16% of the study participants with assistance from community care providers.

We attempted phone interviews for Study B but encountered the following challenges: (a) poor recording clarity over speaker phone, (b) interview fatigue and (c) inability to capture nonverbal cues. Having interval breaks to mitigate fatigue disrupted the flow of conversations. We considered using videoconferencing platforms such as Zoom for focus group discussions (Study C), but concerns about turn taking and lack of suitable online platforms to conduct group modelling activities meant that in-person discussions were the only viable option. Fortunately, our collaborator had a large training room that could accommodate 15 people comfortably while adhering to safe distancing measures.

Studies A, B and C involve participant observation, which requires proximity to observe and interact with older persons in their lived environments. Bodycams were initially considered but vetoed due to concerns about privacy, quality of data and liability issues pertaining to loss/damage of devices.

Study E, which focuses on social isolation, requires older persons to photograph their daily activities and coping strategies for loneliness. We found this activity challenging if conducted via Zoom, as participants would need devices to transfer images, support screen sharing and high-speed Internet. Narrating images in person is a more tactile and interactive experience. Proximity becomes more empowering for the participants, enabling a higher degree of involvement with their photos in interaction with their interviewer (see also [33]).

Research subjects in the community who (a) refused or are ineligible for vaccinations and (b) refrained from wearing masks during interviews called for additional precautions.

Without compromising participants’ comfort and the safety of both parties, research staff would assess risk and keep further distance (e.g. 2 m rather than 1 m safe distancing) from participants, encourage participants to hydrate, provide breaks, practice hand hygiene, sanitize interview materials, and ensure the interview area is well ventilated. Maintaining safe distancing was found to be a challenge, especially when interviewing older persons with hearing impairment. We had to use masks with transparent windows so such participants can participate by lip reading (refer to Additional file 1: Table S5).

## Practicing flexibility and accommodation in research methods with older persons

The psychosocial impact of the pandemic on older persons in Singapore has already been well established in the literature [3], and what we have come to learn is that alternative modes of recruitment and data collection were not feasible despite attempts to modify and adapt according to the situation.

Due to the highly personal and often sensitive nature of our research questions, we found that “traditional” or face-to-face approaches, rather than alternative methods, continue to be preferred. This preference was both expressed by our participants and the research team, who recognized that the nature of the research necessitated a face-to-face approach. A simple transition of data collection methods from in-person to alternative methods such as phone interviews or virtual platforms was assessed as inadequate. A radical reinterpretation and rethinking of the nature of research is needed to ensure that research resilience is built up [10] while concurrently ensuring vulnerable older persons are not left behind in this “new normal” of research design.

However, alternative modes of data collection should not be dismissed entirely. Future cohorts of older persons will be those who are digitally literate and may be accustomed to alternative modes of data collection. Ultimately, research must be adaptable to the evolving needs and demographic features of the population.

The pandemic has necessitated a significant re-evaluation of our research protocols in the following areas:

1. *Importance of rapport building* Pandemic restrictions meant that we had to rely on a single gatekeeper in the form of our collaborator sited within the hospital to help us recruit participants. We had to thus train the gatekeeper to build rapport with potential recruits while recognize that they also face challenges in being part of research during the pandemic. The training involved ensuring that the gatekeepers had access to a script so that they could introduce the research to the older persons in a consistent, digestible manner. When this process was done effectively, they were able to provide both context and content so that by the time we contacted the older persons they were already primed and comfortable to proceed with the study. Our follow-up recruitment call could then be used as an opportunity for us to check in with the older persons on their well-being before we schedule interview sessions as part of our rapport building.
2. *Revising interview questions to make them concise and easy to understand* In the process of making interview questions concise for the phone interview format, we found that the adapted guide also worked well in person. We realized that older persons preferred questions to be short (three to five words), rather than longer sentences. Concurrently, we also found that for our quantitative questionnaire surveys, skip-logic questions had to be streamlined and simplified to prepare for the possibilities of mail-in hard copies or digital responses.
3. *Awareness of complexities associated with longitudinal research* Previously, we relied on existing community partners to refer and maintain contact with participants for follow-up interviews. With COVID-19 adversely impacting these programmes and healthcare providers redeployed to address more urgent pandemic management duties, it was no longer feasible for providers to bridge contact. This led to the realization that we needed to have our own strategies of maintaining relationships with participants in longitudinal research designs. These strategies include re-contacting participants quarterly or doing home visits when we are in the vicinity. However, as we work with transient populations who are subject to many more restrictions during the pandemic in Singapore, we have considered increasing the frequency of check-ins to once a month in order not to lose touch with participants as they might be even more adversely affected by the vagaries of the pandemic.
4. *Adopting creative means to engage potential older persons, especially when there is hesitation to participate in research during a crisis* This could take the form of holding meet-ups, sharing sessions or check-in sessions with participants in our database to inform them of upcoming research opportunities and to gather interest to participate. We also considered utilizing several avenues for recruitment, including virtual Zoom sharing, physical sharing in community places, neighbourhoods and senior activity centres. For a vulnerable population who might prioritize survival more than research participation during the pandemic, it was important to increase modes by which the community can access the research.
5. *Recognizing blind spots that were previously not apparent in usual data collection methods* We found that online platforms cannot be adopted as a direct replacement for offline data collection methods: blind spots such as varying levels of digital literacy, access and adoption need to be considered.
6. *Placing participants’ needs above research key performance indicators* The decision to continue, pause or terminate research projects came with many significant implications and repercussions. Trade-offs must be weighed carefully and calibrated accordingly when considering different research objectives and decisions. Sensitivity to timing is also an important consideration—we could not, for example, embark on the research project centred on loneliness and vulnerability right after the “Circuit Breaker” period, as it might significantly impact the findings with a recency bias. Another adjustment we made is to include interview questions on experiences and needs during the pandemic to capture participants’ existing concerns.

## “New research normal” with older persons: differentiated approaches

Older persons are a vulnerable population at risk of harm and exploitation due to age, and functional and disability status [34]. Hence, research studies with older persons as subjects require conscientious adherence to ethical standards of practice in research [35]. More than ever, the current climate of uncertainty poses an urgent need for researchers to cultivate deeper sensitivities in terms of how the “new research normal” [10] may engender new forms of vulnerabilities and exclusions to an already vulnerable population. Pilbeam et al. discuss how it is more productive to examine categories of vulnerability and areas of sensitivity as interactive and dynamic; changing over the course of a research relationship or a single interview rather than remaining static [36].

From our studies, we observed how the pandemic has heightened vulnerabilities for more groups of older persons, especially given digital exclusion, socioeconomic inequalities, health and/or functional constraints, social isolation, and threats to safety over the course of 2 years. Topics such as social support or perception of health which were usually not perceived as sensitive become more so in a time when risk of infection and mortality increases older persons’ anxieties about whether they are receiving adequate care and have people they can turn to for help during periods of heightened restrictions.

The way forward in the new research normal is to avoid homogenizing the experiences of older persons and to continually review how adaptations include or exclude certain groups of older persons, or how certain challenges may be specific to birth cohorts of older persons. This ensures that ethical standards of justice, autonomy, respect of persons, beneficence and non-maleficence are not compromised [37]. The methods we adopt should not reinforce or exacerbate the sense of exclusion and helplessness that groups or cohorts of older persons already experience.

In this article, we have outlined our reflexive processes in terms of the several strategies and tools that require ongoing adaptation and tailoring as we conduct research in an ever-changing landscape of restrictions. We had to consider the diverse contexts of vulnerability and sensitivity that may emerge over the course of research to address potential risks to both researchers and the older persons involved in our research as subjects. What we learned from the pandemic is that accommodation and flexibility in the form of provisioning diverse tools for outreach and data collection methods needs to be proactively embedded in the research design rather than a reaction to a crisis. Differentiated, rather than standardized, approaches to data collection is the new research normal, including how we approach consent and the supplementary materials required to ensure ease and comfort for participants so that they are open to communicate their needs and participate meaningfully in the research process.

A differentiated approach also compels us to reassess existing hierarchies in research methods, particularly in terms of its implication to social inclusion in research without compromising research rigor, quality and integrity. Adjustments to data collection methods or research design are necessary to ensure that a significant representation of older persons who are eligible to participate in research—voices relevant to the research agenda—are not excluded from participation. Strategies for social inclusion in research methods should align with the objectives of the study and the diverse needs of potential older participants whose views and life experiences warrants representation in the data.

Additionally, it is important to note that the challenges documented by the team, in terms of the digital divide, access and literacy, are particular to the current cohort of older Singaporeans and may not persist beyond the next two decades. In this regard, the lessons we have learned and specific recommendations outlined in this article may not be relevant to future cohorts of older Singaporeans who will be more educated and acquire digital dexterity. Interviews conducted digitally or remotely may alienate current cohorts of older participants, but it may well be the preferred method of conducting interviews for future older cohorts. Thus, researchers need to anticipate the different types of vulnerabilities that current and future cohorts of older persons may experience during the conduct of research and ensure that data collection methods are future-proof so those needs and preferences can be met accordingly.

The COVID-19 pandemic has been a sudden and unprecedented event. Many experienced researchers were unprepared for disruptions at such a massive scale. The pandemic poses new ethical considerations: whose needs to prioritize when considering whether to continue, pause or terminate a research project altogether. As researchers, we are tasked with the mandate to complete deliverables for our funding agencies. At the same time, we grapple with the understanding that, while research findings about social isolation, caregiving and transitional care are important during the pandemic, survival, health and safety are at the forefront of our participants’ concerns. Pushing a research agenda through when these concerns are dominant renders the research tone-deaf. The decision to power through research is not a mere testament to research resilience, but a decision that considers how its continuation maximizes benefit for the community in the long run, with full consideration to ethics amidst heightened existing needs and risks in the evolving COVID-19 pandemic.

## Supplementary Information

**Additional file 1: Table S1.** Nationwide restrictions affecting in-person research. **Table S2.** Chronological list of hospital visitation protocols. **Table S3.** Data collection challenges for quantitative survey (Study F). **Table S4.** Modes of interview for qualitative data collection. **Table S5.** Disease transmission risk and mitigation strategies for conducting interviews using different modes during the pandemic (qualitative study).

## Data Availability

Not applicable. The article discusses research methodologies rather than findings from datasets across Studies A–F.
