# Supplementary material for: Ethics of participation and social inclusion of older persons in research: lessons learned from the COVID-19 pandemic in Singapore
Source: Health Res Policy Syst. 2022 Nov 29;20(Suppl 1):126. doi: 10.1186/s12961-022-00930-2 (PMC9706825; doi:10.1186/s12961-022-00930-2)
Supplement: Supplementary file 1 — Additional file 1: Table S1. Nationwide restrictions affecting in-person research. Table S2. Chronological list of hospital visitation protocols. Table S3. Data collection challenges for quantitative survey (Study F). Table S4. Modes of interview for qualitative data collection. Table S5. Disease transmission risk and mitigation strategies for conducting interviews using different modes during the pandemic (qualitative study). [file 12961_2022_930_MOESM1_ESM.docx]

**Ethics of Participation and Social Inclusion of Older Persons in Research: Lessons learnt from the Covid-19 Pandemic in Singapore**

Ad MAULOD PhD^1^, Sasha ROUSE MA^1^, Atiqah Lee MA^1^, Malcolm RAVINDRAN BNurs^1^, HAZIRAH Mohamad MA^1^, Veronica GOH MPH^3^, DIYANA Azman BSocSc^1^, Lian Leng LOW MBBS MFM MCR^2^, Rahul MALHOTRA MD MPH^1^, and Angelique CHAN PhD^1^

^1^Centre for Ageing Research and Education (CARE), Duke-NUS Medical School

^2^Department of Family Medicine and Continuing Care, Singapore General Hospital

^3^Independent Researcher, formerly affiliated to CARE

Corresponding Author: Ad Maulod ([ad.maulod@duke-nus.edu.sg](mailto:ad.maulod@duke-nus.edu.sg))

**Table S1**

Nationwide restrictions affecting in-person research

| **Type of restriction** | **Description** | **Impact on Research Activities** |
| --- | --- | --- |
| Singapore raising DORSCON level to Orange | Authorities raised DORSCON level to Orange on 7 February 2020. | - No research was conducted from 7 February to 1 June 2020 - No COVID-19 research safety protocols put in place |
| Circuit Breaker | Movement restrictions in Singapore from 7 April 2020 to 1 June 2020. | - No research was conducted at this point - Non-essential activities and social interactions outside of one’s household were not allowed |
| Limit on household visitors | The prevailing limit of either 2 or 5 unique visitors per household per day. | - Most participants in our target population (older persons in Singapore) receive essential visitors per day (e.g., family members helping to care for them, or community providers who visit to provide care), leaving little room for researchers to visit - Difficult to schedule an interview with participants with higher needs - Significant delay on the data collection timeline |
| Limit on household visits | The prevailing limit of either 1 or 2 household visits per day per interviewer. | - Interviewers who were able to conduct 4 interviews per day pre-pandemic were now limited to 1 or 2 per day - Significant extensions to the data collection timeline |
| Limit on group sizes | For research, the prevailing limit of 5 individuals, safe distanced at 1 metre apart applies. For trainings, up to 50 individuals were allowed to be in groups of 5, safe distanced at 1 metre apart. | - Few venues accessible for research allow for safe distancing - Any group-related research (e.g., focus group discussions, participant review gatherings) which exceeded 5 participants (including members of the research team) had to be postponed |

**Table S2**

*Chronological list of hospital visitation protocols*

| **Hospital Visitation Protocols** | **Impact on recruitment of older persons** |
| --- | --- |
| When Singapore raised DORSCON level to Orange (7 February 2020), no visitors were allowed except for deathly ill patients | - No recruitment conducted in the wards |
| During Circuit Breaker (April 2020 to June 2020), no visitors were allowed except for deathly ill patients | - No recruitment conducted in the wards |
| From June 2020 to October 2020, visitors were allowed for patients. No external research staff allowed to visit patients. | - No recruitment conducted in the wards |
| In October 2020, the hospital allowed external researchers using N95 masks allowed in wards. Before resuming face-to-face interactions, the research team must follow strict safety protocols and receive approval from the hospital. | - Recruitment resumed in December 2021.  - Eligible pool of participants decreased significantly, affecting recruitment rates |
| In April 2021, only hospital staff were allowed to have facetime with patients. | - Two-month hold in recruitment  - Difficulties finding dedicated hospital staff:  ·     Manpower restrictions due to COVID deployment  ·     Researchers trained hospital staff to conduct recruitment and obtain approval from the hospital. |

**Table S3**

*Data collection challenges for quantitative survey (Study F)*

| **Alternative mode** | **Challenges** | **Additional tasks to rectify challenges (research team)** |
| --- | --- | --- |
| Online | - Incomplete survey submitted - Vague or unclear responses to open-ended questions - Time to complete survey varies as participants can pause and continue later - A lot of “refused” or “don’t know” responses - There is no control over the identity of the person who completes the questionnaires as family members or friends not recruited in the study may have helped with the responses | - Sent multiple reminders for participants to complete survey - Called participants to verify vague or unclear responses |
|  |  |  |
|  |  |  |
| Voice Call | - Participants may not answer an unfamiliar number due to fear of fraud calls - Heavy reliance on tone of voice (i.e., lack of visual cues) to gauge participants’ understanding of questions or reaction to sensitive questions(e.g., “How satisfied as you with your sex life?”) | - Manually entered responses from voice call onto an online survey platform (i.e., Qualtrics) |
| Video Call | - (For those without webcam) Heavy reliance on tone of voice (i.e., lack of visual cues) to gauge participants’ understanding of questions or reaction to sensitive questions(e.g., “How satisfied are you with your sex life?”) | - Manually entered responses from video call onto Qualtrics |
| Mailed-in survey questionnaire | - Incomplete survey questionnaires - There is no control over the identity of the person who completes the questionnaires as family members or friends may have helped with the responses - Participants may take up to a few months to return completed questionnaire | - Customised questionnaire as skip logic was too difficult for participants to comprehend (e.g., if “no” is answered, skip to Section X) - Manually entered responses from mailed-in questionnaires onto Qualtrics - Called participants to check on completion and mail status - ·   Called participants to verify vague or unclear responses |

**Table S4**

*Modes of interview for qualitative data collection*

| **Data collection method** | **Description** | **Modes of interview** |
| --- | --- | --- |
| In-depth interviews (Longitudinal)     - **Study B** - **Study G** | Baseline and follow-up interviews. For Study B, interviews were conducted prior to the start of the programme, and after the programme has concluded. For Study G, interviews were conducted at baseline and 12-months post baseline. | - In-person (Default) - Voice call (alternative) - Video call (alternative) |
| In-depth interviews (Cross-sectional)     - **Study A** - **Study C** - **Study D** - **Study E** | Interviews conducted at one specific time point, no follow-up interviews | - In-person (Default) - Video call (alternative) |
| Photo Elicitation Interview     - **Study A** - **Study E** | After participants complete a 2-week photo journaling activity, the interviewer will ask questions based on the photos taken | - In-person (Default) - Video call (alternative) |
| Participant observations     - **Study A** - **Study B** - **Study C** | Single interactions where researchers document (including spatial mapping, photography, video- and audio-recording) observations of participants in their environment | - In-person - No alternative offered |
| Focus Group Discussions, with group and model building activities     - **Study C** | Interviews conducted with a group of participants. Group activities include mapping a wide array of presented challenges to arrive to solutions. | - In-person - No alternative offered |

**Table S5**

Disease transmission risk and mitigation strategies for conducting interviews using different modes during the pandemic (Qualitative Study)

| **Task** | **Challenges & risk of disease transmission** | **Strategies to mitigate risks** |
| --- | --- | --- |
| **Scheduling interviews** | **Over-the-phone (low risk)**   - Researchers are informed when participants may have had a change in COVID-19 status, without physical contact   **In-person (high risk)**   - Home visits required to schedule interviews with referred participants who are not contactable - No way to find out if participant is an active COVID-19 case prior to visit, or issued a health risk warning (HRW) | **Over-the-phone**   - Call to schedule interview 7-12 days prior to intended interview date - Remind participants of scheduled interview 2-3 days prior and conduct COVID-19 safety checks - Call again on the day of interview and conduct COVID-19 safety checks - Reschedule interviews (14 days later) for those who are active COVID-19 cases, or a close contact   **In-person**   - Mask-on all times, practice hand hygiene - Ring doorbell/ knock on door and then stand 1 metre away when waiting for a response - Conduct COVID-19 safety checks- If safe to enter, sanitise hands prior to entering household, offer participant a surgical mask and practice safe distancing |
| **Conducting In-depth interviews remotely** | **Videoconferencing (low risk)**   - Challenge to obtain virtual/verbal consent due to diverse profiles of older persons - Access to internet connection that can support video-conferencing platforms | **Videoconferencing**   - Digital consent forms available for tech-savvy participants via hyperlink - For participants who can do videoconferencing, but do not know how to navigate digital consent (e.g., e-signatures) verbal consent must be sought prior to recording. Verbal consent will be audio-recorded - All recordings to be saved and uploaded to an encrypted cloud server and delete the local copy |
| **Conducting in-person data collection: In-depth interviews; Photo elicitation interviews; Participant observations;** **Focus group discussions** | **In-person (medium to high risk)**   - Participants may unknowingly be asymptomatic COVID-19 cases | **In-person**   - Mask-on always for both interviewer and participant, practice hand hygiene and safe distancing - Interview venue must be well-ventilated - Ensure participant’s hands are sanitised before handling any items (e.g., documents, pen, tablet) - Sanitise all common touchpoints after each interview (e.g., pen, tablet) - For households: Ensure participants will not expect more than the stipulated amount of unique household visitors after accounting for the interviewers |
